# Reporting rates of abnormal uterine bleeding after COVID-19 vaccination in South Korea

**DOI:** 10.3389/fdsfr.2025.1658412

**Published:** 2025-11-14

**Authors:** Jae Min Lee, Seung Geun Yeo, Sung Soo Kim, Dong Choon Park

**Affiliations:** 1 Department of Otorhinolaryngology Head & Neck Surgery, College of Medicine, Kyung Hee University Medical Center, Seoul, Republic of Korea; 2 Medical Research Center for Bioreaction to Reactive Oxygen Species and Biomedical Science Institute, Core Research Institute (CRI), Kyung Hee University, Seoul, Republic of Korea; 3 Department of Biochemistry and Molecular Biology, College of Medicine, Kyung Hee University, Seoul, Republic of Korea; 4 Department of Obstetrics and Gynecology, St. Vincent’s Hospital, College of Medicine, The Catholic University of Korea, Seoul, Republic of Korea

**Keywords:** COVID-19 vaccination, uterine bleeding, adverse event, women’s health, vaccine safety

## Abstract

**Introduction:**

Abnormal uterine bleeding after COVID-19 vaccination has been reported, but few studies have characterized population-level reporting rates. We evaluated AUB reporting in South Korea.

**Methods:**

We analyzed suspected adverse reactions reported to the Korea Disease Control and Prevention Agency from 26 February 2021 to 29 June 2023 (week 121). Cases with AUB were identified, and reporting rates were compared across vaccine types and doses.

**Results:**

During the study period, 135,894,788 doses were administered, with 483,391 suspected adverse reactions (356 per 100,000) and 6,288 AUB reports (4.9 per 100,000). AUB ranked 15th among reported adverse events. Reporting rates varied by manufacturer and dose.

**Discussion:**

These descriptive pharmacovigilance findings do not estimate incidence or imply causality. Prospective, controlled studies are needed to clarify causal relationships and to inform diagnostic and management protocols for AUB after vaccination.

## 1 Introduction

The World Health Organization (WHO) has estimated that the global fatality rate of patients infected with the SARS-CoV2 virus during the COVID-19 global pandemic was about 2.1%, but had decreased to about 0.9% as of 6 August 2023 (World Health Organization, 2023). This fatality rate varies greatly by country and age, with older age, immune compromised status, and underlying diseases being associated with severe illness and death. Vaccination against COVID-19 has been shown to reduce the risk of infection, as well as to reduce rates of severe illness and death. In general, immunity to the virus after vaccination requires about 2 weeks, during which time individuals can become infected with the virus (Korean Society of Internal Medicine Standard Practice Guideline Committee, 2021; Center for Disease Control and Prevention, 2021).

Vaccines can have unexpected adverse reactions, however, even when administered appropriately. The most common adverse reactions following COVID-19 vaccination include fever, chills, muscle pain, fatigue, pain at the injection site, fever, swelling, itching, headache, nausea, vomiting, abdominal pain, and rash (Korea Disease Control and Prevention Agency, 2021; Kim et al., 2021). Because the benefits of COVID-19 vaccination clearly outweigh the risks of side effects, vaccination is recommended unless there are special circumstances. Although women may experience abnormal uterine bleeding after COVID-19 vaccination, few studies have analyzed this adverse reaction (Dellino et al., 2022; Laganà et al., 2022; Paik and Kim, 2023). The present study therefore analyzed the reporting rate of abnormal uterine bleeding following COVID-19 vaccination reported to the Korea Centers for Disease Control and Prevention. Korea is comprised of a relatively unique ethnic group and is a government-led vaccination country. The administered types of vaccine are limited and the vaccination protocol is consistent. In addition, the management and distribution of vaccines is carried out according to a certain manual and is unified accordingly, making it very meaningful as a sample group. In this study, based on the reporting rate of abnormal uterine bleeding after COVID-19 vaccination reported in the Korea Centers for Disease Control and Prevention, we reported the frequency of abnormal uterine bleeding as an adverse reaction after COVID-19 vaccination and presented a possible mechanism.

## 2 Materials and methods

The database of the Korea Centers for Disease Control and Prevention was reviewed to determine the rates of adverse reactions after COVID-19 vaccination from 26 February 2021 (week 0) to 29 June 2023 (week 121). Adverse reactions were defined as undesirable and unintended signs, symptoms, or diseases occurring after vaccination. A causal relationship between adverse reactions and vaccines was not necessary, and adverse reactions were not data that proved the adequacy of the diagnosis or causality by the vaccine (Korea Disease Control and Prevention Agency, 2021). Adverse events were reported as suspected adverse reactions after vaccination against COVID-19 and were calculated based on the reports by medical institutions in South Korea. These results are reported by the medical institution that performed the vaccination after individual patients self-defined abnormal uterine bleeding after vaccination with COVID-19 vaccines. Minor reactions were those not requiring hospitalization or significant medical intervention (e.g., low-grade fever, fatigue, injection-site pain). Major reactions were those requiring hospitalization, causing significant disability, or life-threatening conditions. Participants were categorized as experiencing abnormal uterine bleeding if they reported symptoms to medical institutions after COVID-19 vaccination. These reports were subjective and based on each individual’s perception of abnormal bleeding. Data regarding the number of people who received COVID-19 vaccines and any subsequent adverse reactions were collected from the website of the Korea Centers for Disease Control and Prevention (https://ncv.kdca.go.kr/eng/), which updates these data weekly. Five COVID-19 vaccines were administered to individuals throughout Korea: AZD1222 (AstraZeneca), BNT162b2 (Pfizer-BioNTech), JNJ-78436735 (Janssen), mRNA-1273 (Moderna), and NVX-CoV2373 (Novava. The Janssen vaccine was administered via a one-dose regimen, whereas the other four vaccines were administered via a two-dose regimen. Vaccination data, including vaccine type, epidemiological data, symptom onset dates, symptoms, and complications from the Korea Centers for Disease Control and Prevention, were collected (Korea Disease Control and Prevention Agency, 2023; Kim et al., 2023). We summarized death reports by demographics, reported causes, and time-to-event where available. Given frequent missingness in passive reports, most cases were unassessable for causality; counts and reporting rates were interpreted descriptively. The study protocol was approved by the Institutional Review Board of Kyung Hee University Hospital, which waived the requirement for informed consent due to the retrospective nature of this study (IRB No. 2022-06-042).

## 3 Results

### 3.1 Analysis of adverse reactions following COVID-19 vaccination

**FIGURE 1:**
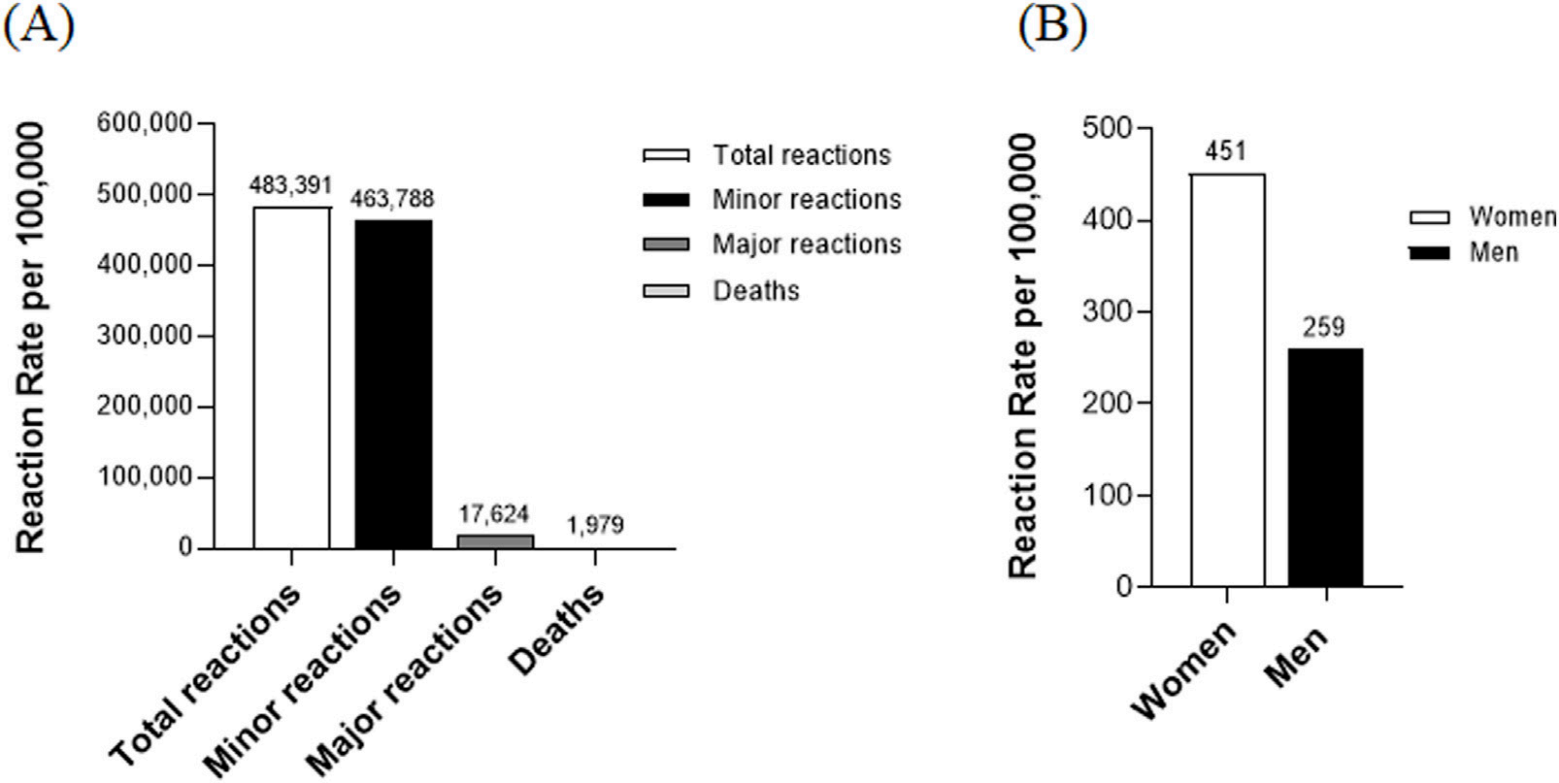
Analysis of adverse reactions following COVID-19 vaccination. **(A)** Distribution of adverse reaction types: This figure illustrates the distribution of adverse reaction types following COVID-19 vaccination. Minor reactions: The number of cases with minor adverse reactions. Major reactions: The number of cases with major adverse reactions. Deaths: The number of death cases reported post-vaccination. **(B)** Adverse reaction rates by gender: This section of the figure analyzes adverse reaction rates based on gender.

During the study period, a total of 135,894,788 doses of COVID-19 vaccine were administered, with suspected adverse reactions reported following 483,391 doses, equating to 356 adverse reactions per 100,000 vaccinations. Among these 483,391 adverse events, the majority, 463,788 (95.9%), were classified as minor. In contrast, 17,624 (3.6%) were considered major, and 1,979 death reports (0.4% of all reports) were submitted following vaccination (Figure 1A). These are counts from a passive surveillance system and do not imply causality. Cause-of-death fields were not systematically available in the KDCA passive reports; therefore, we were unable to provide a breakdown of reported causes. The reporting rate of adverse reactions was approximately 1.7 times higher in women compared to men, with reporting rates of 451 and 259 per 100,000 vaccinations, respectively (Figure 1B).

### 3.2 Age-related variability in adverse reaction rates to COVID-19 vaccination

**FIGURE 2:**
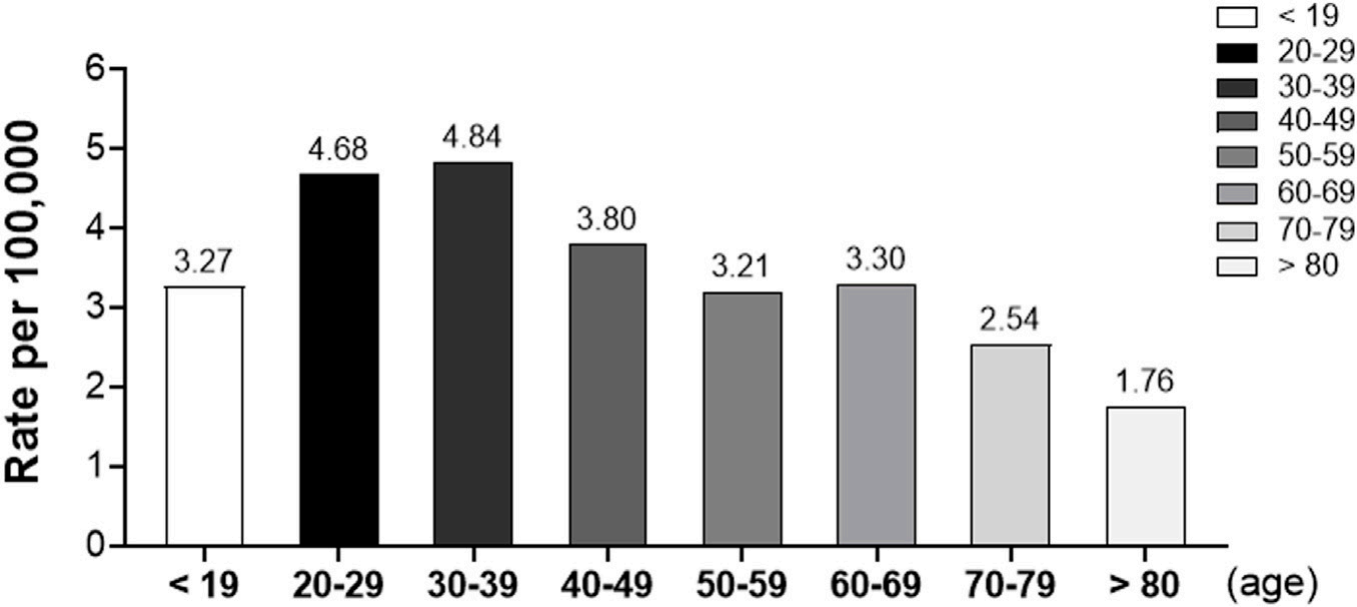
Rates of adverse reactions by age group following COVID-19 vaccination. This bar graph illustrates the reporting rates of suspected adverse reactions per 100,000 vaccinated individuals across different age groups.

When analyzed by age group, the rates of adverse reactions were highest in subjects aged 30–39 (4.84 per 100,000) and 20–29 (4.68 per 100,000), somewhat high in subjects aged 40–49 (3.80 per 100,000), 60–69 (3.30 per 100,000), ≤19 (3.27 per 100,000), and 50–59 (3.21 per 100,000) years, and lowest in subjects aged 70–79 (2.54 per 100,000) and ≥80 (1.76 per 100,000) years. The highest rates were observed in the 30–39 and 20–29 age groups, with rates of 4.84 and 4.68 per 100,000, respectively. The rates decreased progressively in older age groups, with the lowest rate observed in individuals aged 80 and above, at 1.76 per 100,000. This data highlights age-related variations in the reporting rate of adverse reactions following vaccination (Figure 2).

### 3.3 Comparative analysis of adverse reaction rates across different COVID-19 vaccine doses and types

**FIGURE 3:**
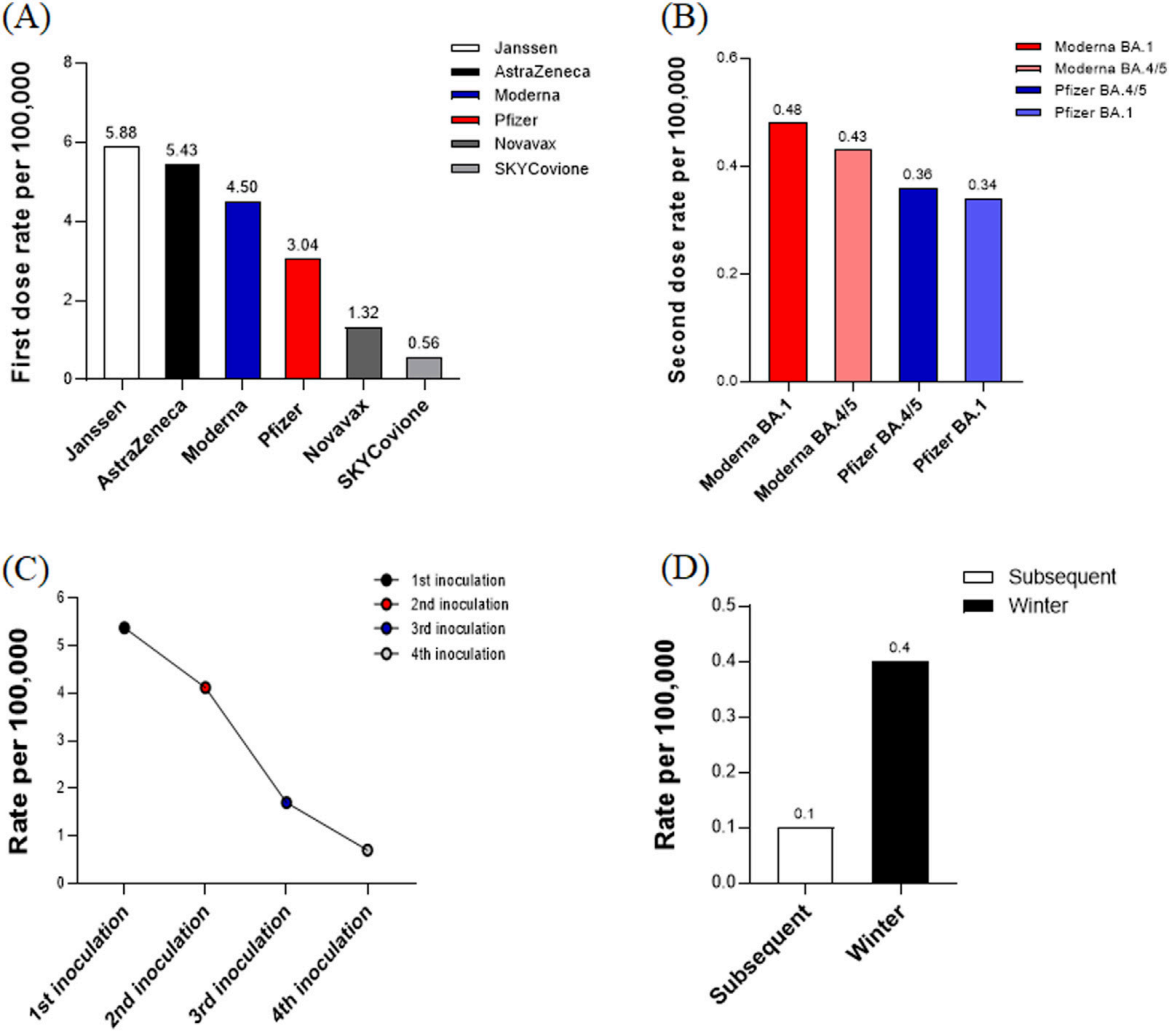
**(A)** Adverse reaction rates for first dose by vaccine manufacturer. This bar chart illustrates the adverse reaction rates per 100,000 vaccinations for different COVID-19 vaccines after the first dose. The reaction rates are highest for Janssen and AstraZeneca and lowest for SKYCovione, indicating variability in adverse reaction frequency across different manufacturers. **(B)** Adverse reaction rates for seconddose by vaccine manufacturer. This bar chart shows the adverse reaction rates per 100,000 vaccinations for bivalent COVID-19 vaccines after the second dose. The reaction rates are relatively low across all manufacturers, with the highest rate observed for Moderna (BA.1). **(C)** Overall adverse reaction rates by dose number. This line graph illustrates the overall adverse reaction rates per 100,000 vaccinations across the first, second, third, and fourth doses of COVID-19 vaccines. **(D)** Adverse reaction rates for subsequent doses and winter administrations. This bar chart displays the adverse reaction rates per 100,000 vaccinations for subsequent doses and winter administrations of COVID-19 vaccines.

The rates of adverse reactions following the administration of the first doses of various monovalent COVID-19 vaccines were as follows: Janssen (5.88 per 100,000, 8,893/1,511,664), AstraZeneca (5.43 per 100,000, 110,486/20,348,870), Moderna (4.50 per 100,000, 113,296/25,156,452), Pfizer (3.04 per 100,000, 246,782/81,182,703), Novavax (1.32 per 100,000, 1,282/972,694), and SKYCovione (0.56 per 100,000, 3/5,310). Notably, Janssen and AstraZeneca vaccines exhibited the highest rates of adverse reactions, while SKYCovione had the lowest, indicating variability in reaction frequencies across different manufacturers (Figure 3A). For the second doses of bivalent vaccines, the adverse reaction rates were 0.48 per 100,000 for Moderna (BA.1), 0.43 for Moderna (BA.4/5), 0.36 for Pfizer (BA.4/5), and 0.34 for Pfizer (BA.1). All manufacturers reported relatively low reaction rates, with the highest rate observed for Moderna (BA.1) (Figure 3B). Overall, the adverse reaction rates after the first, second, third, and fourth doses were 5.38, 4.12, 1.70, and 0.70 per 100,000, respectively. This data demonstrates a decreasing trend in adverse reaction rates as the number of doses increases, suggesting a reduction in the frequency of side effects with additional inoculations (Figure 3C). For subsequent doses and winter administrations, the rates were 0.10 and 0.40 per 100,000, respectively. These figures indicate a lower rate of adverse reactions for subsequent doses compared to winter administrations, underscoring the continued decline in reaction rates with further doses. This trend suggests that the vaccine’s safety profile remains robust even as administrations extend beyond the initial doses (Figure 3D).

### 3.4 Rates and types of suspected adverse reactions by symptom in COVID-19 vaccine recipients

**FIGURE 4:**
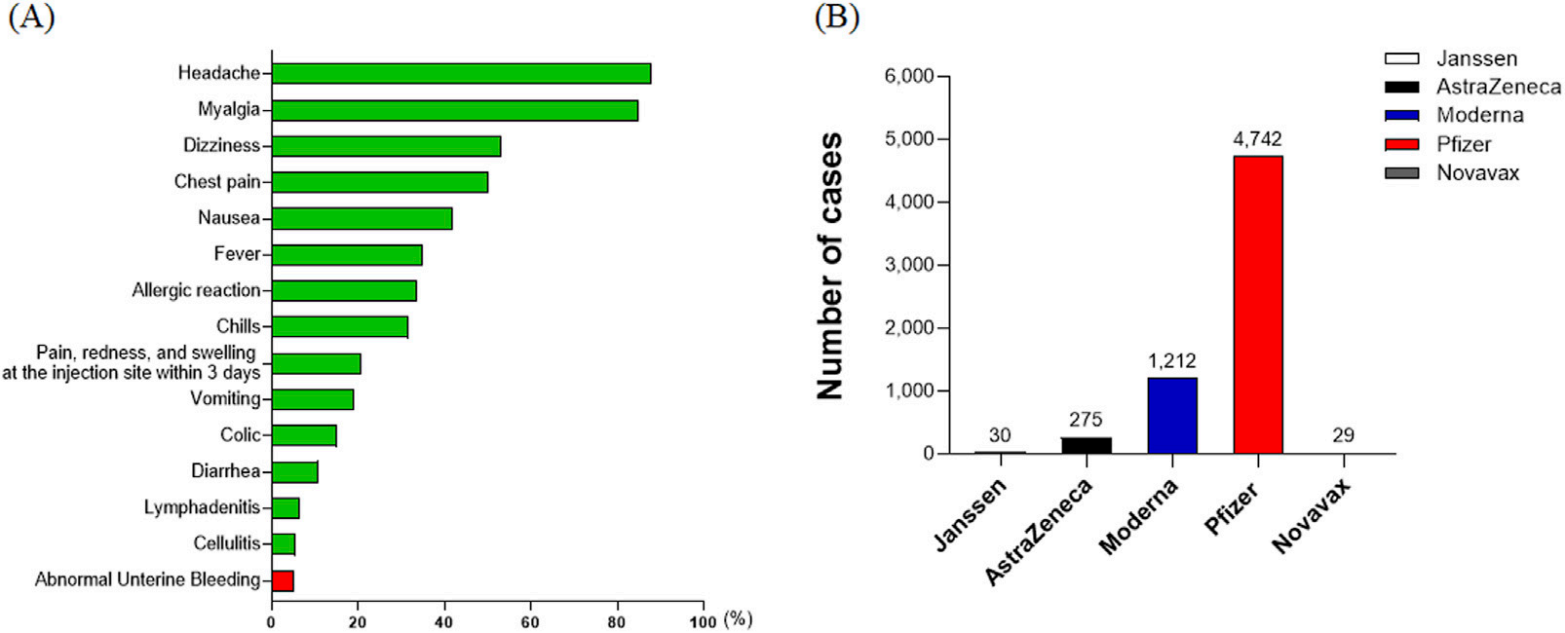
Rates and types of suspected adverse reactions by symptom in COVID-19 vaccine recipients. **(A)** The ranking of abnormal uterine bleeding as the 15th most common adverse event among COVID-19 vaccine recipients. The condition was reported at a rate of 4.9 per 100,000 vaccinated women. **(B)** Distribution of 6,288 reported cases of abnormal uterine bleeding by vaccine type. The majority of these cases were associated with the Pfizer vaccine, indicating it had the highest number of reported adverse reaction cases in this category.

Among the suspected adverse reactions to the COVID-19 vaccine, abnormal uterine bleeding was identified as the 15th most common adverse event (Figure 4A). This condition was reported following the administration of 6,288 vaccine doses, with a rate of 4.9 per 100,000 vaccinated women. Breaking down these 6,288 incidents of abnormal bleeding by vaccine type, the majority of cases, 4,742 (75.41%), were associated with the Pfizer vaccine, indicating it had the highest number of reported adverse reaction cases in this category. This was followed by 1,212 cases (19.27%) with Moderna, 275 cases (4.37%) with AstraZeneca, 30 cases (0.48%) with Janssen, and 29 cases (0.46%) with Novavax. It is important to note that the number of women vaccinated with each vaccine varied significantly, and therefore, these figures should not be interpreted as a direct comparison of the adverse event rates between vaccines (Figure 4B).

## 4 Discussion

We deliberately prioritized abnormal uterine bleeding among the many adverse events reported after COVID-19 vaccination for several reasons. First, abnormal uterine bleeding poses a significant clinical and psychosocial burden, often leading to healthcare utilization, thus making its even low absolute rates meaningful at the population level. Second, routine pharmacovigilance has consistently highlighted a prominent and recurring signal of post-vaccination menstrual complaints, which warrants focused examination beyond anecdotal reports. Third, transient post-vaccination immune activation and cytokines can modulate hypothalamic–pituitary–ovarian signaling and endometrial innate immunity, both of which are known to influence menstrual timing and flow, while rare immune-mediated thrombocytopenia could also affect bleeding patterns. We do not claim causality, but these mechanisms justify focused characterization of abnormal uterine bleeding. Lastly, South Korea’s government-led vaccination program with standardized logistics and limited vaccine platforms provides a robust setting for platform-stratified reporting rate estimates. Our aim was to characterize the signal to inform prospective studies and clinical protocols, acknowledging that causal inference cannot be established from passive surveillance.

Normal menstruation varies considerably among individuals, making it difficult to define abnormal uterine bleeding. Nevertheless, bleeding that deviates from normal menstrual patterns can be collectively defined as abnormal. In normal menstruation, the luteal phase is relatively constant, with an average of 14 days, but there may be fluctuations in the follicular phase (Norwitz ER, 2013a). In contrast, the type of abnormal uterine bleeding may vary according to the duration or volume of bleeding, with causes differing by age. Abnormal bleeding may be accompanied by organic lesions, but may also be associated with emotional disorders, mental stress, drug administration, blood disorders, systemic disorders, and nutritional disorders (Norwitz ER, 2013b). Abnormal uterine bleeding reported after COVID-19 vaccination can range from amenorrhea to excessive uterine bleeding, and clinical management generally depends on the underlying cause. In many women, however, the cause of abnormal uterine bleeding after COVID-19 vaccination cannot be easily determined. In clinical practice, pelvic ultrasonography is often used to exclude structural pathology when abnormal uterine bleeding is reported. However, the KDCA passive surveillance dataset used in this study does not capture standardized follow-up testing (e.g., estrogen profiles or pelvic ultrasonography) or adjudicated diagnostic outcomes; therefore, we cannot determine how many women underwent these evaluations or were found to have no pathological findings within our cohort. Accordingly, statements regarding ultrasound monitoring and spontaneous resolution in the absence of pathology reflect observations from prior reports and clinical practice rather than case-level data from our dataset. We explicitly acknowledge these constraints among the study’s limitations, including the self-defined nature of abnormal uterine bleeding reports, lack of standardized validation, and the descriptive design based on passive surveillance.

COVID-19 vaccines can be classified as nucleic acid (messenger RNA) vaccines, viral vector (adenovirus) vaccines, inactivated vaccines, and recombinant vaccines (COVID-19 Vaccination Response Promotion Team, 2021; Korean Society of Internal Medicine Standard Practice Guideline Committee, 2021). Vaccines induce biological reactions that differ from those of chemical substances, such that the type and reporting rate of vaccine side effects may vary depending on the manufacturer, even for the same vaccine, and batch number, even for the same manufacturer. Because of these differences in biological responses to vaccines, rare side effects may occur at any time. Surveillance systems are therefore needed to monitor adverse reactions after vaccination. Although evidence of induction or causality is required to determine whether symptoms that occur after vaccination are side effects of the vaccine, causality is generally difficult to determine (COVID-19 Vaccination Response Promotion Team, 2021; Son et al., 1994). Epidemiological data are therefore required to evaluate the comparative risk of developing the same abnormal symptom in vaccinated and unvaccinated groups.

Three items are required to determine whether a side effect is caused by a vaccine. First, it is necessary to determine the minimum potential possibility that a vaccine can cause side effects at a certain rate under certain circumstances. Therefore, side effects rates should be compared in vaccinated and unvaccinated individuals, followed by a determination of the biologic plausibility of these side effects and the association of adverse reactions with vaccine components. Second, retrospective causality should be assessed to determine whether vaccines cause side effects in vaccine recipients. Third, predictive causality should be determined by assessing the likelihood that people vaccinated with the same vaccine will show the same side effects, at the predicted frequency, in the future (Institute of Medicine, 1990).

In South Korea, uterine bleeding was the 15th most frequently reported adverse event of COVID-19 vaccination, occurring after administration of 4.9 per 100,000 doses. The pathophysiology of abnormal uterine bleeding after COVID-19 vaccination is unclear, but several potential mechanisms have been suggested. Although the effects of the COVID-19 vaccine are generally of short duration, the long-lasting impact of chronic SARS-COV-2 infection could potentially render the menstrual cycle prone to abnormalities (Male, 2021). Stress and psychological pain itself are among the causes of menstrual abnormalities, suggesting that menstrual irregularities may be due to the stresses of the COVID-19 pandemic, not the vaccination itself. Similar to COVID-19 vaccine administration, an intense immune response following infection with SARS-COV-2 may also alter the hypothalamic-pituitary-ovarian axis (Turnbull and Rivier, 1999; Skelly et al., 2021; Girardi and Bremer, 2022). The specific antigen that triggers this immune response remains unclear, although the COVID-19 spike protein and the adjuvant sucedin are possible candidates. The COVID-19-associated spike protein is located on the surface of the SARS-CoV-2 virus, and all vaccines generate neutralizing antibodies to this protein, activating immune cells to prevent or mitigate future SARS-CoV-2infection (Heinz and Stiasny, 2021). Cyclic breakdown and restoration of the uterine endometrium are mediated by innate immune cells residing in the endometrium (Monin et al., 2020). Activation of these immune cells could be responsible for heavier, irregular, and untimely bleeding. Hormone levels involved in the menstrual cycle could also be affected by vaccine-induced immunological changes, including but not limited to thyroid abnormalities, which could also disrupt menstrual cycles. Post-vaccination menstrual changes may also be caused by immune-mediated vaccine-induced thrombocytopenia, which has been observed following administration of several other vaccines, such as those for measles, hepatitis, and diphtheria (Williams et al., 2007; Perricone et al., 2014; Malik et al., 2021).

The post-vaccine immune response stressor has been hypothesized to affect the development of the dominant follicle during the follicular phase, which would, in turn, affect the length of the menstrual cycle (Barbarino et al., 1989). Retrospective studies also found that estradiol-containing contraceptives reduced the likelihood that women would experience post-vaccination menstrual disturbances (Alvergne et al., 2022; Lee et al., 2022). Estrogen and estradiol have anti-inflammatory and immunomodulatory properties, protecting against severe COVID-19-relatedclinicaloutcomes (Al-kuraishy et al., 2021; Mauvais-Jarvis et al., 2020). Large numbers of post-vaccination variations in cycle length were observed in women who received two doses of mRNA COVID-19 vaccines within a single menstrual cycle. Inactivated viral particle vaccines may therefore be a safer alternative for women with previous menstrual abnormalities. To date, therefore, all major studies have found that COVID-19 vaccinations have only mild and temporary effects, with no long-term clinical consequences. Although reports have described abnormal uterine bleeding after COVID-19 vaccination, it is difficult to establish a connection or analyze the exact mechanism, as diagnostic criteria for causal relationships have not yet been established.

Accurate identification of abnormal uterine bleeding after COVID-19 vaccination requires several considerations. First, it is necessary to determine the presence or absence of underlying diseases to evaluate whether the bleeding is newly caused by the vaccine or an exacerbation of pre-existing conditions. Additionally, the type, timing, and frequency of the vaccine administered during the menstrual cycle should be assessed. Objective indicators, such as hormonal changes and antibody titers, are essential for diagnosing abnormal uterine bleeding post-vaccination. Furthermore, prospective, large-scale studies are needed to clarify the relationship between vaccines and abnormal uterine bleeding.

The present study provides valuable descriptive data on the reporting rate of abnormal uterine bleeding following COVID-19 vaccination. During the study period, 1,979 death reports were submitted following COVID-19 vaccination in the passive surveillance dataset. These are all-cause death reports temporally associated with vaccination and should not be interpreted as evidence of causality. Cause-of-death information and clinical details were variably reported and frequently incomplete, which limited formal causality assessment for most cases. Potential underreporting, duplicate reports, and lack of standardized validation further constrain interpretation. Accordingly, death report counts and any derived reporting rates are descriptive pharmacovigilance metrics rather than incidence or risk estimates. However, several limitations should be noted. First, abnormal uterine bleeding after vaccination was self-defined by individuals and recorded by reporting institutions without standardized diagnostic adjudication or objective test confirmation; therefore, misclassification is possible. Second, because the analysis relies on passive surveillance data, causal inference is not feasible, and reporting bias (under- or selective reporting) may affect observed rates. Third, although the KDCA dataset includes case-level reporting, it does not provide sufficiently granular or consistently complete temporal information (e.g., menstrual cycle day at onset, standardized follow-up), limiting our ability to evaluate symptom timing relative to vaccination and, by extension, biological plausibility. Fourth, key potential confounders—such as age, contraceptive use, and prior gynecologic conditions—were not available for adjustment in our descriptive analysis, which may influence the observed reporting rate of abnormal uterine bleeding. Finally, our analysis was purely descriptive and did not include inferential statistics or control groups, which limits quantitative uncertainty assessment and causal interpretation. To address the limitations of passive surveillance and lack of standardized validation, future studies should use prospective or active-surveillance designs with standardized case definitions, cycle-level timing, and detailed covariates. Employing appropriate comparators (e.g., vaccinated vs. unvaccinated or self-controlled designs) and adjusted analyses will improve causal assessment and refine risk estimates.

## 5 Conclusion

In conclusion, within passive surveillance data from South Korea, abnormal uterine bleeding had a reporting rate of 4.9 per 100,000 administered doses among women and ranked 15th among reported adverse events. These descriptive findings inform pharmacovigilance signal characterization but do not estimate incidence or imply causality; prospective, controlled studies are needed to refine risk assessment.

## Data Availability

The dataset analyzed in this study is subject to privacy and confidentiality restrictions and is available to qualified researchers who comply with the Korea Disease Control and Prevention Agency (KDCA) data use policies. Access requires a formal request and approval from the KDCA. Guidance and contact information for data access are provided on the KDCA website. For further information, please contact the corresponding author at yeo2park@gmail.com.

## References

[B1] Al-kuraishy H. M. Al-Gareeb A. I. Faidah H. Al-Maiahy T. J. Cruz-Martins N. Batiha G. E.-S. (2021). The looming effects of estrogen in Covid-19: a rocky rollout. Front. Nutr. 8 (March 18), 649128. 10.3389/fnut.2021.649128 33816542 PMC8012689

[B2] Alvergne A. Woon E. Von and Male V. (2022). Effect of COVID-19 vaccination on the timing and flow of menstrual periods in two cohorts. Front. Reproductive Health 4 (July 25), 952976. 10.3389/frph.2022.952976 36303656 PMC9580734

[B3] Barbarino A. Marinis L. D. Folli G. Tofani A. Casa S. D. D’Amico C. (1989). Corticotropin-Releasing hormone inhibition of gonadotropin secretion during the menstrual cycle. Metabolism 38 (6), 504–506. 10.1016/0026-0495(89)90208-4 2498612

[B4] Center for Disease Control and Prevention (USCDC) (2021). Interim clinical considerations for use of COVID-19 vaccines currently authorized in the United States.

[B5] COVID-19 Vaccination Response Promotion Team (2021). Coronavirus-19 vaccination business guidelines for hospital-level or higher medical institutions (Self-Vaccination).

[B6] Dellino M. Lamanna B. Vinciguerra M. Tafuri S. Stefanizzi P. Malvasi A. (2022). SARS-CoV-2 vaccines and adverse effects in gynecology and obstetrics: the first Italian retrospective study. Int. J. Environ. Res. Public Health 19 (20), 13167. 10.3390/ijerph192013167 36293746 PMC9603573

[B7] Girardi G. Bremer A. A. (2022). Scientific evidence supporting coronavirus disease 2019 (COVID-19) vaccine efficacy and safety in people planning to conceive or who are pregnant or lactating. Obstetrics and Gynecol. 139 (1), 3–8. 10.1097/AOG.0000000000004636 34727554 PMC8678336

[B8] Heinz F. X. Stiasny K. (2021). Distinguishing features of current COVID-19 vaccines: knowns and unknowns of antigen presentation and modes of action. Npj Vaccines 6 (1), 104. 10.1038/s41541-021-00369-6 34400651 PMC8368295

[B9] Institute of Medicine (1990). Report of committee to review and epidemiologic study of neurologic illness and vaccination in children. Washington DC.

[B10] Kim H. J. Kim H. H. Park G. Y. Kang D. Y. (2021). Precautions for COVID-19 vaccination. Pharmacoepidemiol Risk Manag. 13, 38–44. 10.56142/2021.13.1.38

[B11] Kim S. H. Yon D. K. Choi Y. S. Lee J. Park K.-H. Lee Y. J. (2023). Vertigo and dizziness after coronavirus Disease-2019 vaccination: a nationwide analysis. J. Int. Adv. Otology 19 (3), 228–233. 10.5152/iao.2023.22937 37272641 PMC10331640

[B12] Korea Disease Control and Prevention Agency. (2021). Who gets the Covid-19 vaccine first?

[B13] Korea Disease Control and Prevention Agency. (2023). COVID-19 vaccination adverse case status report.

[B14] Korean Society of Internal Medicine Standard Practice Guideline Committee (2021). Korean society of internal medicine COVID-19 vaccine guideline version 1.0. Korean Soc. Intern. Med.

[B15] Laganà A. S. Veronesi G. Ghezzi F. Ferrario M. M. Cromi A. Bizzarri M. (2022). Evaluation of menstrual irregularities after COVID-19 vaccination: results of the MECOVAC survey. Open Med. Wars. Pol. 17 (1), 475–484. 10.1515/med-2022-0452 35350834 PMC8919838

[B16] Lee K. M. N. Junkins E. J. Luo C. Fatima U. A. Cox M. L. Clancy K. B. H. (2022). Investigating trends in those who experience menstrual bleeding changes after SARS-CoV-2 vaccination. Sci. Adv. 8 (28), eabm7201. 10.1126/sciadv.abm7201 35857495 PMC9286513

[B17] Male V. (2021). Effect of COVID-19 vaccination on menstrual periods in a retrospectively recruited cohort.

[B18] Malik J. Malik A. Javaid M. Zahid T. Ishaq U. Shoaib M. (2021). Thyroid function analysis in COVID-19: a retrospective study from a single center. PLOS ONE 16 (3), e0249421. 10.1371/journal.pone.0249421 33784355 PMC8009384

[B19] Mauvais-Jarvis F. Klein S. L. Levin E. R. Estradiol P. (2020). Estradiol, progesterone, immunomodulation, and COVID-19 outcomes. Endocrinology 161 (9), bqaa127. 10.1210/endocr/bqaa127 32730568 PMC7438701

[B20] Monin L. Whettlock E. M. Male V. (2020). Immune responses in the human female reproductive tract. Immunology 160 (2), 106–115. 10.1111/imm.13136 31630394 PMC7218661

[B21] Norwitz ER S. J. (2013a). Obstetrics and gynecology guidelines and overview, 4th ed. Chapter 03. Reproductive physiology. Wiley-Blackwell.

[B22] Norwitz ER S. J. (2013b). Obstetrics and gynecology guidelines and overview, 4th ed. Chapter 11. Abnormal uterine bleeding. Wiley-Blackwell.

[B23] Paik H. Kim S. K. (2023). Female reproduction and abnormal uterine bleeding after COVID-19 vaccination. Clin. Exp. Reproductive Med. 50 (2), 69–77. 10.5653/cerm.2023.05925 37258099 PMC10258518

[B24] Perricone C. Ceccarelli F. Nesher G. Borella E. Odeh Q. Conti F. (2014). Immune thrombocytopenic purpura (ITP) associated with vaccinations: a review of reported cases. Immunol. Res. 60 (2–3), 226–235. 10.1007/s12026-014-8597-x 25427992

[B25] Skelly D. T. Harding A. C. Gilbert-Jaramillo J. Knight M. L. Longet S. Brown A. (2021). Two doses of SARS-CoV-2 vaccination induce robust immune responses to emerging SARS-CoV-2 variants of concern. Nat. Commun. 12 (1), 5061. 10.1038/s41467-021-25167-5 34404775 PMC8371089

[B26] Son Y. M. (1994). Analysis of vaccine side effects and causal relationships. Korean J. Pediatr. 4 (1), 15–22.

[B27] Turnbull A. V. Rivier C. L. (1999). Regulation of the hypothalamic-pituitary-adrenal axis by cytokines: actions and mechanisms of action. Physiol. Rev. 79 (1), 1–71. 10.1152/physrev.1999.79.1.1 9922367

[B28] Williams N. I. Berga S. L. Cameron J. L. (2007). Synergism between psychosocial and metabolic stressors: impact on reproductive function in cynomolgus monkeys. Am. J. Physiology-Endocrinology Metabolism 293 (1), E270–E276. 10.1152/ajpendo.00108.2007 17405827

[B29] World Health Organization (2023). WHO coronavirus (COVID-19) dashboard. Available online at: https://covid19.who.int.

